# About a Peculiar Form of Senegalese *Polycarpon* (Caryophyllaceae) and a Diagnostic Key to the African Taxa in the Genus

**DOI:** 10.3390/plants15071007

**Published:** 2026-03-25

**Authors:** Duilio Iamonico

**Affiliations:** Department of Environmental Biology, University of Rome Sapienza, Piazzale Aldo Moro 5, 00185 Rome, Italy; duilio.iamonico@uniroma1.it

**Keywords:** Africa, new combination, *Polycarpon prostratum* var. *littorale*, *Polycarpon tetraphyllum s.l.*, Senegal

## Abstract

As part of an ongoing investigation into the genus *Polycarpon*, the name *P. prostratum* var. *littorale*, described from Senegal in 1967 by J. Raynal and A. Raynal, was studied. It does not appear in *World Flora Online* (WFO), is unplaced in *Plant Of the World Online* (POWO) and in the *International Plant Names Index* (IPNI). Furthermore, no study appears to be published after that by J. Raynal and A. Raynal. With the aim to clarify the identity and taxonomic status of Raynal & Raynal’s variety, a detailed study of the type and other specimens is here presented. The name is lectotypified on a specimen preserved at P (barcode P00388964) and further two isolectotypes were traced (P00388965 and P00388966). Based on the morphology of Raynal & Raynal’s taxon and the current circumscription of the genus *Polycarpon* as monophyletic (with *P. tetraphyullum*) a nomenclatural change (*P. tetraphyllum* subps. *littorale comb. et stat. nov.*) is proposed. The subspecies is assessed as Endangered based on the IUCN criterion B2. Furthermore, a diagnostic key to the *Polycarpon* taxa occurring in Africa is presented.

## 1. Introduction

The genus *Polycarpon*, as traditionally circumscribed, would include about 16 species distributed in tropical and temperate regions of the world [1,2]. Kool et al. [3], by using DNA sequence data from the chloroplast rps16 intron and nuclear RPB2 regions, demonstrated the polyphyly of this genus and highlighted three different clades with high support values, i.e., (1) the *P. coquimbense*/*suffruticosum* aggregate (from South America), (2), the *P. tetraphyllum* aggregate (main diversity in the Mediterranean region), and (3) *P. prostratum* (Forssk.) Asch. & Schweinf. (tropical, widespread). The first clade was excluded from *Polycarpon* since its members lack the synapomorphy for this genus, i.e., the capsule dehiscence by valves that elastically roll inwards. Iamonico [4] transferred *P. coquimbense* Gereau & Martic. and *P. suffruticosum* Griseb., together with *P. anomalum* Hassl., to the new genus *Augustea* Iamonico, endemic from South America (Argentina, Bolivia, Chile, and Paraguay); two years after Iamonico [4], Iamonico & Montesinos-Tubée [5] also recombined *P. moreiranum* Muñoz-Schick under *Augustea*. As regards the second clade *sensu* Kool et al. [3] (the *P. tetraphyllum* aggregate), it represents a polyploid complex that can be treated as a single species, *P. tetraphyllum* (L.) L. [3,4]; accordingly, it is proposed to recognize the various *taxa* at subspecies rank (see e.g., [6] and literature therein). Finally, concerning *P. prostratum* (Forssk.) Asch. & Schweinf., its identity is clarified in my own study (currently under submission) where it was verified that Forsskål’s *Alsine prostrata* (basionym of *P. prostratum*) is actually a heterotypic synonym of *Pharmaceum depressum* L. and a new combination (subspecific rank) under *Polycarpon teraphyllum* is proposed (in the present paper I preliminary accept Forsskål’s taxon as a separate species according to [7]).

Among the remaining names in *Polycarpon* there is *P. prostratum* var. *littorale* J. Raynal & A. Raynal, described from Senegal [8], which appears unplaced in POWO [9], without original publication in IPNI ([10], as “Unknown Publ.”), and even it does not appear in WFO [7]. Thus, Raynal & Raynal’s variety needs clarification about its taxonomic status, and this issue is addressed in the present paper as part of an ongoing investigation on the genus *Polycarpon* in the world (see, e.g., [7] and literature therein).

## 2. Materials and Methods

The work is based on analysis of relevant literature and examination of specimens preserved at the herbaria B, BR, C, IFAN, K, P, and US (acronyms according to Thiers [11]). Nomenclature articles cited throughout the text follow the *International Code of Nomenclature for algae, fungi, and plants* (hereafter reported as “ICN”), in its current *Madrid Code* edition [12].

The area of Occupancy (AOO) and Extent of Occurrence (EOO) were calculated based on the known collections by using GeoCAT [13]. Assessment of the risk of extinction is based on the IUCN criteria and categories [14].

## 3. Results and Discussion

### 3.1. Typification of Polycarpon prostratum var. littorale

Raynal and Raynal [8] (pp. 310–311) validly described *Polycarpon prostratum* var. *littorale* as a different white-tomentose form of *P. prostratum* with perennial habit, ovate to obovate leaves (3 mm × 2 mm), and rose flowers; the type was reported as follows: “Holotypus: J. & A. Raynal 6058, in depressionibus humidis inter arenas mobiles propre pagum vulgo Kayar dictum ad litorem septentrionalem Promontorii Viridis, 7-7-1960, P!”. I traced three specimens at P (barcodes MNHN-P-P00388964, MNHN-P-P00388965, and MNHN-P-P00388966) which are duplicates of the same collection (no. 6058) by J. Raynal and A. Raynal on 7 July 1960 at “bord secs d’une dépression interdunaire humide (partie non inondable) … Kayar (Senegal), cordon de dunes vives limitant au NW le Tanma” as reported in the original labels. Since Raynal and Raynal [8] did not specify which of the three aforementioned specimens is the holotype, they inadvertently cited syntypes (see Arts. 9.2 Note 1 and 9.6 of ICN). Consequently, a lectotype should be designated according to the Arts. 9.3 and 9.4 of ICN. I here propose the specimen MNHN-P-P00388964, which is the more complete, bearing many leaves, flowers, and fruits, features which are important in the taxonomy of the genus *Polycarpon* [1,2]. The other two specimens (MNHN-P-P00388965 and MNHN-P-P00388966) are isolectotypes.

It is to be noted that, after Raynal and Raynal [8], *Polycarpon prostratum* var. *littorale* was cited only one time in published works, i.e., in *Plantas Vasculares e Briófitos da Guiné-Bissau* [15] (p. 43), where the variety was firstly recorded in Guinea-Bissau at Bijagós archipelag and, doubtfully in the Southern Region.

### 3.2. Taxonomic Treatment

***Polycarpon tetraphyllum*** subsp. ***littorale*** (J. Raynal & A. Raynal) Iamonico, *comb. et stat. nov.* ***≡*** *Polycarpon prostratum* var. *littorale* J. Raynal & A. Raynal, Adansonia, n.s., 7: 310. 1967.

Lectotype (here designated):—SENEGAL. Thiès, Kayar, bord secs d’une dépression interdunaire humide (partie non inondable), 7 July 1960, *J. Raynal et A. Raynal 6058* (MNHN-P-P00388964!, image of the lectotype available at https://plants.jstor.org/stable/viewer/10.5555/al.ap.specimen.p00388964?loggedin=true&loggedin=true accessed on 9 March 2026); isolectotypes: MNHN-P-P00388965 (image at https://plants.jstor.org/stable/viewer/10.5555/al.ap.specimen.p00388965?loggedin=true accessed on 9 March 2026), and P00388966 (image at https://plants.jstor.org/stable/viewer/10.5555/al.ap.specimen.p00388966?loggedin=true accessed on 9 March 2026).

**Figure 1:**
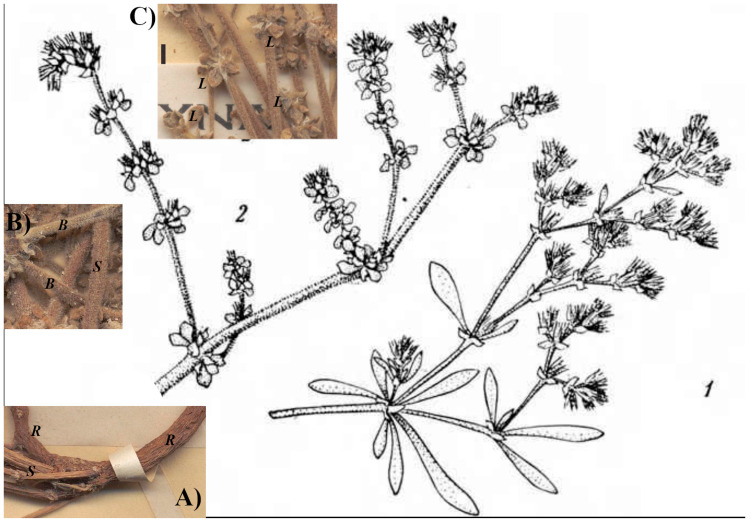
*Polycarpon tetraphyllum* subsp. *littorale* (J. Raynal & A. Raynal) Iamonico: illustration by Raynal and Raynal [8] (p. 310, Figure 2: “1”, var. *prostratum*; “2” var. *littorale*) with details [from the lectotype MNHN-P-P00388964; image credit: Muséum national d’Histoire naturelle (MNHN) – Paris Herbarium (P)] of the diagnostic characters: (**A**) perennial root (“R”), (**B**) white-tomentose stem (“S”) and branches (“B”), (**C**) minute and ovate leaves (“L”; scale bar: 2 mm).

**Diagnosis** (Figure 1)**:** *Polycarpon tetraphyllum* subsp. *littorale* differs from *Polycarpon prostratum* by the white-tomentose pubescence on all parts of the plant (*vs*. glabrous or pubescent, but never tomentose), perennial habit (hemicryptophyte *vs*. therophyte), leaves ovate to obovate, 3 mm × 2 mm [*vs*. obovate to spathulate, (3–)5–15(–25) × (0.3–)1.5–2.5(–5) mm], and rose flowers (vs. white).

**Description:** Plant perennial (hemicryptophyte); stem prostrate-diffuse, 25–35 cm tall, white-tomentose; leaves mostly in whorl of four, ovate to obovate, small [(4–)3.5–3.0(–2.5) × (0.5–)1.5–2.0(–2.5) mm], white-tomentose, base attenuate, apex acute; stipules scarious, triangular to lanceolate, acute to acuminate, 1–2(–3) mm long; inflorescences in dense cymes both axillary and terminal; flowers sessile (rarely very shortly peduncled); sepals ovate, 2.5–3.0(–3.5) mm long, not keeled, apex acute (sometime mucronate), margin hyaline (each hyaline side up to 1/3 about wide than the total width of the sepal); petals 5, membranous, entire, shorter than the sepals (1.0–1.5 mm long); stamens usually 3, shorter than the sepals; capsule ovoid (2.0–2.5 mm long), slightly shorter than the sepals; seed brown, reticulate, ca. 0.5 mm in diameter.

**Etymology:** The species epithet refers to the habitat (coastal dunes).

**Proposed vernacular name:** Kayar allseed.

**Phenology:** Two phenological periods can be considered, the first flowering period ranges from November to May (dry season), the second fruiting period ranges from June to October (rainy season).

**Distribution and habitat:** *Polycarpon tetraphyllum* subsp. *littorale* is restricted to a few localities of western Senegal and at Bijagós archipelago in Guinea-Bissau; the occurrence in the South region of Guinea-Bissau [15] (p. 43) remains in doubt, since no specimen was traced confirming this site. The subspecies grows mainly on active coastal dune, at near sea level.

**Figure 2:**
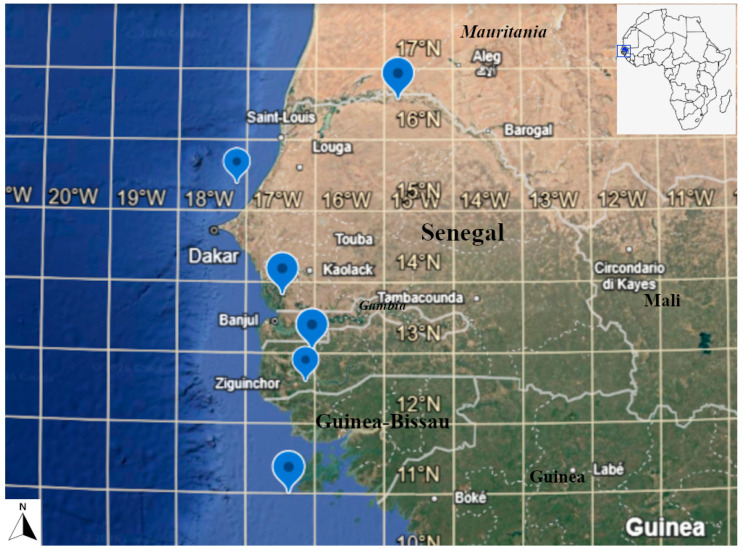
Distribution map (blue placemarks) of *Polycarpon tetraphyllum* subsp. *littorale* (J. Raynal & A. Raynal) Iamonico.

**Conservation status:** At the current state of knowledge, the occurrence of *Polycarpon tetraphyllum* subsp. *littorale* is restricted few localities of western Senegal and at Bijagós archipelago in the Atlantic Ocean (Guinea-Bissau) (Figure 2). Based on these localities (see “Other Specimens Examined”), AOO (Area of Occupancy) is 24,000 km^2^, while EOO (Extend of Occurrence) is 58,751 km^2^. According to the criterion B1 (based on AOO) of IUCN [14], *P. tetraphyllum* subsp. *littorale* is assessed as Least Concerned (LC), whereas by applying criterion B2 (based on AOO), the taxon is assessed as Endangered (EN). Main threat is represented by tourism. Based on the principle of precautionary, the higher category of risk is here proposed, i.e., Endangered.

**The genus *Polycarpon* in Africa:** according to *African Plant Database* [16], *Polycarpon tetraphyllum* occurs in Africa with six subspecies [subsp. *alsinifolius* Biv.) Ball., subsp. *catalunicum* (O. Bolòs & Font Quer) Iamonico & Domina, subsp. *diphylum* (Cav.) O. Bolòs & Font Quer, subsp. *herniarioides* (Ball) Iamonico & Domina, subsp. *polycarpoides* (Biv.) Iamonico; *sensu* Iamonico and Domina [2] *plus* the one [subsp. *littorale* (J. Raynal & A. Raynal) Iamonico] here proposed (reported in SANBI [16] as *P. prostratum* var. *littorale*) and *P. prostratum* (Forssk.) Asch. & Schweinf. (my paper is under submission to clarify the correct name and rank to use for this taxon). A first diagnostic key to the *Polycarpon* taxa occurring in Africa is below presented (*Polycarpon prostratum* is here preliminary accepted as separate species according to [16]).

1 Plants annual (therophytes) ....................................................................................................... 2

– Plants perennial (hemicryptophytes) ....................................................................................... 4

2a. Leaves fleshy, at least some purplish or reddish; seeds smooth…………………………………………………...... *P. tetraphyllum* subsp. *alsinifolius*

– Leaves not fleshy, green; seeds verrucose or tuberculate ……………………………......... 3

3a. Flowers arranged in lax cymes; sepals short (less than 1 mm), acute to acuminate and keeled; fruit up to 2 mm long …………………………. *P. tetraphyllum* subsp. *tetraphyllum*

– Flowers arranged in dense cymes; sepals 1–2 mm long, obtuse and not keeled; fruit up to 2–2.5 mm long ……………………………………………………… *Polycarpon prostratum*

4a. Leaves small (up to 6 mm long), appearing in two types of phyllotaxy (opposite the lower leaves, in whorl of four the upper ones) ……… *P. tetraphyllum* subsp. *herniarioides*

– Leaves 10–15 mm long on average (rarely shorter than 10 mm), in one type of phyllotaxy (opposite or in whorl of four) ………………………………………………………………. 5

5a. Leaves opposite ……………………………………….. *P. tetraphyllum* subsp. *polycarpoides*

– Leaves arranged in whorl of four …………………………………………………………..... 6

6a. Plants white-tomentose pubescence on all parts of the plant; leaves ovate to obovate, 3 × 2 mm; flowers rose ………………………………………… *P. tetraphyllum* subsp. *littorale*

– Plants glabrous or pubescent (never tomentose); leaves obovate to spatulate, (3–)5–15(–25) × (0.3–)1.5–2.5(–5) mm; flowers white ……………. *P. tetraphyllum* subsp. *tetraphyllum*

**Other Specimens Examined:**

***Polycarpon tetraphyllum* subsp. *littorale*.** SENEGAL. Podor: Guédé, *s.d.*, leg. *Decourtis 7*, det. *U. G. Adam 20 June 1959* (IFAN; IFAN25807!); Thiès: Lac Tanma, 21 October 1968, *J. Berhaut 7490* (BR0000070005752!); Fatick: Toubakouta, 6 m, 10 August 1977, *C. Vanden Berghen 1971* (BR0000014022616!); *ibidem*, *C. Vanden Berghen 1962* (BR0000014022623!); Ziguinchor, Agnak, 5 m, 7 August 1972, *C. Vanden Berghen 5416* (BR0000014022586!); Petit Koulaye, 5 m, 24 August 1979, *C. Vanden Berghen 3470* (BR0000014022593!).

***Polycarpon prostratum.*** BENIN. Colline, Ouèssè, Idadjo, Galeria ferestière, 22 March 2001, *A. Akoègninou 2347* (BR0000014022821!). CENTRAL AFRICAN REPUBLIC. Bozoum, Oubangui Chari, 01 May 1933, *G. Le Testa 3314* (BR0000014022951!). CHAD. Chari Central, 25-30 June 1903, *A. Chevalier 9161* (BR0000014022890!). GUINEA-BISSAU. Aluviones humidas, 1955, *E. S. Sousa 2063* (B-10-0757866!). EGYPT. Cairo, *P. Forsskål 559* (C10001619!). INDIA. Bengal, circa Calcuttam, 1836, *J. W. Helfer 171* (BR0000028360124!); West Bengal, Kooch Behar, April 1941, *S. K. Mukherjee 441* (US03627448!); *N. Wallich 6962* (K001126381!). MALAWI. Central Region, Kasungu, road to Lingadzi, 1000 m, 22 June 1970, *R. Brummitt 11645* (BR0000009389892!). MALI. Saint-Martin, Marigot, 29 June 1899, *C. Chevalier 1112* (BR0000014022647!). MOZAMBIQUE. Gorongosa National Park, sandy banks of Urema River, 12 October 2015, *J. Guyton et B. Würsten JAG_15_699* (BR0000027333532V!). NIGER. Kollo, Namarc, Piste Niamey, 17 October 1957, *N. Leman 242BIS* (BR0000014022845!). NIGERIA. Borno, border of Lake Alo, 22 June 1982, *E. Denys 730* (BR0000014022869!). ZIMBABWE. Umuguza Reserve, 16 November 1953, *H. Wild 4150* (BR0000014023057!); Pine Tree Inn, Juliasdale, woodland, 16 January 2014, *P. Ballings & B. Würsten 1990* (BR0000027311981V!).

## Data Availability

The original contributions presented in this study are included in the article. Further inquiries can be directed to the corresponding author.
